# Allocentric spatial learning and memory deficits in Down syndrome

**DOI:** 10.3389/fpsyg.2015.00062

**Published:** 2015-02-16

**Authors:** Pamela Banta Lavenex, Mathilde Bostelmann, Catherine Brandner, Floriana Costanzo, Emilie Fragnière, Giuliana Klencklen, Pierre Lavenex, Deny Menghini, Stefano Vicari

**Affiliations:** ^1^Laboratory for Experimental Research on Behavior, Institute of Psychology, University of LausanneLausanne, Switzerland; ^2^Department of Neuroscience, Bambino Gesù Children’s HospitalRome, Italy

**Keywords:** hippocampus, episodic, memory, capacity, resolution, genetic disorder

## Abstract

Studies have shown that persons with Down syndrome (DS) exhibit relatively poor language capacities, and impaired verbal and visuoperceptual memory, whereas their visuospatial memory capacities appear comparatively spared. Individuals with DS recall better where an object was previously seen than what object was previously seen. However, most of the evidence concerning preserved visuospatial memory comes from tabletop or computerized experiments which are biased toward testing egocentric (viewpoint-dependent) spatial representations. Accordingly, allocentric (viewpoint-independent) spatial learning and memory capacities may not be necessary to perform these tasks. Thus, in order to more fully characterize the spatial capacities of individuals with DS, allocentric processes underlying real-world navigation must also be investigated. We tested 20 participants with DS and 16 mental age-matched, typically developing (TD) children in a real-world, allocentric spatial (AS) memory task. During local cue (LC) trials, participants had to locate three rewards marked by local color cues, among 12 locations distributed in a 4 m × 4 m arena. During AS trials, participants had to locate the same three rewards, in absence of LCs, based on their relations to distal environmental cues. All TD participants chose rewarded locations in LC and AS trials at above chance level. In contrast, although all but one of the participants with DS exhibited a preference for the rewarded locations in LC trials, only 50% of participants with DS chose the rewarded locations at above chance level in AS trials. As a group, participants with DS performed worse than TD children on all measures of task performance. These findings demonstrate that individuals with DS are impaired at using an AS representation to learn and remember discrete locations in a controlled environment, suggesting persistent and pervasive deficits in hippocampus-dependent memory in DS.

## INTRODUCTION

### DOWN SYNDROME: GENERAL COGNITIVE PROFILE AND SMALL-SCALE SPATIAL CAPACITIES

Down syndrome (DS) is the most common genetic cause of intellectual disability, with an incidence of 1 in 625–1,000 live births (Bittles et al., 2006; Weijerman et al., 2008). It results from the presence of a partial or complete triplication (trisomy) of chromosome 21. Adults with DS have IQs ranging from 30 to 70 and a typical mental age (MA) ranging from 5 to 9 years of age (Vicari et al., 2005, 2006). Traditionally, individuals with DS were considered to have uniform intellectual impairments affecting all cognitive domains. However, a number of studies, which compared the capacities of individuals with DS to those of individuals with Williams Syndrome and other intellectual disorders, led to the identification of a unique DS cognitive profile (Bihrle et al., 1989; Wang and Bellugi, 1994; Jarrold et al., 1999, 2007; Vicari et al., 2005, 2006; Porter and Coltheart, 2006; Vicari and Carlesimo, 2006; Bussy et al., 2011; Carney et al., 2013). For example, persons with DS exhibit relatively poor language capacities, with impairments in receptive and expressive language (Chapman et al., 1991; Chapman, 1997), and specific difficulties in syntactical processing (Abbeduto et al., 2003, 2007). Impaired verbal short-term memory (Jarrold and Baddeley, 1997), especially for phonological information (Raitano Lee et al., 2010), might underlie certain aspects of these language deficits. Individuals with DS also exhibit a global processing bias, neglecting internal details when reconstructing stimuli in the block-design task or in the Delis (Navon) hierarchical processing task (Bellugi et al., 1999). In contrast, individuals with DS show relatively preserved visuospatial memory when asked to recall where objects previously appeared within a display placed directly in front of them. For example, they perform similar to MA-matched typically-developing (TD) controls on the Corsi block-tapping task (Wang and Bellugi, 1994; Jarrold and Baddeley, 1997; Numminen et al., 2001; Laws, 2002) when stimuli are presented sequentially (but not necessarily when visuospatial stimuli are presented simultaneously (Lanfranchi et al., 2009; Yang et al., 2014)). Finally, in a study comparing visual object versus visuospatial memory, individuals with DS exhibited better recall for the previously seen position of an object on a sheet of paper (*where)*, than for the form of the object that was previously seen (*what*; Vicari et al., 2005).

Perhaps because performance on the small-scale visuospatial tasks described above is relatively preserved in individuals with DS, few studies have investigated their large-scale spatial memory capacities. However, performance on small-scale spatial tasks does not necessarily correlate with or predict performance on large-scale spatial tasks in which participants must move around (Quaiser-Pohl et al., 2004; Hegarty et al., 2006; Farran et al., 2010). We describe below why the inherent nature of small-scale visuospatial tasks makes them inappropriate for predicting performance on real-world orientation and navigation tasks, and why, in order to define a comprehensive cognitive profile of DS, it is fundamental to investigate the capacities of individuals with DS in large-scale, allocentric spatial (AS) memory tasks.

### DIFFERENCES BETWEEN VISUOSPATIAL MEMORY AND ALLOCENTRIC SPATIAL MEMORY

The importance and utility of using visuospatial paradigms to study visuospatial memory cannot be overstated. However, visuospatial memory is just one component in the broader domain of spatial memory, and indeed its contribution to solving spatial tasks involving navigation in the real-world is not entirely clear. Although visuospatial tasks are indeed spatial in the sense that participants must localize targets, the fact that the vast majority of visuospatial memory tasks are conducted on a computer screen, or on a piece of paper presented in front of a participant (i.e., “desktop” or “pencil and paper” tasks) limits their generalizability to the broader domain of spatial memory in several key ways.

First, experiments in rodents, monkeys, and humans have yielded consistent results suggesting that there are multiple types of spatial knowledge, and that objects and locations in the environment can be defined with respect to distinct frames of reference [see (Burgess, 2006) for a review]. An egocentric spatial frame of reference defines locations with respect to their position relative to one’s body, in a viewpoint-dependent manner. Thus, locations can be on one’s right, one’s left, behind or in front of one. When navigating, the route to a destination can be encoded as a sequence of landmarks and egocentric turns (e.g., from the hotel, go straight to the convenience store on the corner, turn right, walk two blocks, the restaurant is on the left). In contrast, an AS frame of reference defines locations with respect to their position relative to other objects or locations in the environment, in a viewpoint-independent manner, allowing the construction of a cognitive map of one’s environment (Tolman, 1948). Thus, for example, a speaker’s lecturn is both in front of the audience and on the left of the projection screen, and will maintain these same relations irrelevant of where an observer is standing in the lecture hall. When navigating, a destination encoded in allocentric coordinates is defined by its relations with multiple other locations in the environment, thus allowing the navigator to arrive at the desired destination using even a novel, never-before experienced path.

Second, it is critical to realize that AS memory is not limited to the processing of information from any one sensory modality, such as vision. Instead, AS memory is dependent on the integration of information derived from all sensory modalities, including primarily visual, vestibular, and proprioceptive information, but also auditory, olfactory and somatosensory information. In this manner, AS memory may exist in absence of vision: allocentric representations persist when individuals are physically removed from the target location so that it cannot be seen, when individuals navigate in the dark (Quirk et al., 1990; Save, 1997), and in blind individuals (Passini and Proulx, 1988; Loomis et al., 1993). Thus, while visuospatial information may normally contribute to building an allocentric representation of the environment, visual information is not processed independently from other sensory information (Etienne and Jeffery, 2004). Accordingly, the response properties of place cells (i.e., neurons in the hippocampal formation that encode spatial locations in an allocentric frame of reference) are less specific when only visual information is available, as compared to when coherent visual, vestibular and proprioceptive information is available (Matsumura et al., 1999; Ravassard et al., 2013), but, critically, have been shown to maintain their location-selective firing properties in blind rats (Save et al., 1998).

Finally, whereas large-scale spatial tasks can be used to assess either egocentric spatial memory processes or AS memory processes, highly controlled large-scale spatial tasks are best suited to assess AS memory. Indeed, small-scale visuospatial tasks which are administered directly in front of stationary subjects likely implicate egocentric processes preferentially [but see (Wang and Simons, 1997; Simons and Wang, 1998; Burgess et al., 2004; Nardini et al., 2006; Banta Lavenex et al., 2011), for visuospatial tasks in which the experimental apparatus and/or the participants move]. In contrast, large-scale spatial tasks in which subjects must move around, and in which egocentric strategies are precluded (by eliminating landmarks that can be directly associated with goal locations and by having participants solve the task from multiple starting locations), can better assess AS capacities, and can serve as a specific assay for hippocampus-dependent memory function. Indeed, although the hippocampal formation is known to be critical for episodic and relational memory functions in humans (Scoville and Milner, 1957; Cohen and Eichenbaum, 1993), its role in AS memory has been the most studied and is the best understood across species, from rats, to monkeys, to humans (O’Keefe and Dostrovsky, 1971; O’Keefe and Nadel, 1978; Morris et al., 1982; Banta Lavenex et al., 2006, 2014).

### LARGE-SCALE SPATIAL CAPACITIES IN DOWN SYNDROME

To date, only one study investigated real-world, large-scale spatial capacities in DS. In his doctoral thesis, Mangan conducted spatial memory experiments designed to study the *response learning*, *cue learning*, and *place learning* capacities of children with DS and chronologically age-matched TD children from 16 to 28 months of age (Mangan, 1992). He used a modified version of the holeboard apparatus originally designed for studying AS memory in rats, which consisted of a round platform (3.65 m in diameter) containing 11 symmetrically arranged holes that could hide rewards. The response learning task was an egocentric task that required children to always turn in the same direction on the platform to find the reward; for example, after watching the reward being hidden in one of the four holes surrounding the center hole (and always the same hole for any given child), the child was moved to the center of the platform. From here, if they turned to the right, for example, they would always find the rewarded hole. The cue task could be solved by visual guidance (a non-spatial strategy) since the rewarded hole was always covered by a uniquely colored lid; for example, after watching the reward being hidden in one of the four holes surrounding the center hole and then covered by, for example, the yellow lid, the child was placed in a randomly selected position on the platform. If the child then localized the hole covered by the yellow lid, they would always find the rewarded hole. For both the response task and the cue task, although children with DS needed more trials than TD children to solve the task, they were nonetheless able to find the reward in a final probe trial (PT), following a 1-min delay between when the object was hidden and when the child was allowed to search. In contrast, the performance of children with DS was reported to differ significantly more from that of TD children on the place learning task, a task which requires AS processing. In this task, after watching the reward being hidden (in one of the four holes surrounding the center hole, and always the same hole for any given child), children were started from a different pseudo-randomly chosen location on the outside edge of the platform for every trial. Children needed to use an allocentric representation to identify the location of the rewarded hole, i.e., the position of the rewarded hole relative to distal environmental cues in the room. As for the response and cue learning tasks, children with DS required more trials than TD children to learn the place learning task. However, during the PT (after 1 min between hiding and searching) children with DS did not focus their search at the goal location, and instead searched locations surrounding their start location on the outside edge of the platform, suggesting memory impairments specific to AS memory processes. Nevertheless, because Mangan (1992) studied the spatial abilities of very young (16–28 month-old) children with DS, his results might be inconclusive. Basic AS memory capacities do not emerge in TD children until around 24 months of age (Newcombe et al., 1998; Ribordy et al., 2013), and even then allocentric processes are far from mature (Ribordy et al., 2013; Ribordy Lambert et al., accepted). This leaves the possibility that the development of AS memory processes is only delayed in young individuals with DS, and that they may continue to develop normally, albeit with a slower time course than in TD children. To address this question the AS capacities of fully developed individuals with DS must be assessed.

### DS SPATIAL CAPACITIES IN VIRTUAL ENVIRONMENTS

Three studies investigated the spatial capacities of individuals with DS in virtual environments and found their performance impaired. In theory, virtual environments are designed to emulate real-world large-scale environments, and therefore should be able to test AS abilities (but see below). In a first study, to determine whether cognitive deficits seen in DS were more specifically consistent with dysfunction associated with the hippocampus or the prefrontal cortex, Pennington et al. (2003) tested mature participants with DS [mean chronological age (CA): 14.7 years] and MA-matched TD children (mean CA: 4.9 years) on a battery of neuropsychological tests designed to asses the function of these two cortical regions. One of the tasks was a virtual Morris water maze. In the real-world Morris water maze used with rodents, the animals’ ability to find an invisible platform slightly submerged under water is impaired by hippocampal damage (Morris et al., 1982). In Pennington’s study, children had to learn the position of a “rug” in the middle of a virtual room containing distal visual cues such as a door, a picture frame, etc. During a 90-s PT (the only data reported for this task), participants with DS spent less time searching in the correct quadrant than TD children. Overall, participants with DS exhibited worse performance than TD children on a battery of tasks evaluating hippocampal function. In contrast, in tasks evaluating prefrontal cortex function, participants with DS did not differ from MA-matched children (Pennington et al., 2003).

In a second study, Courbois et al. (2013) investigated the wayfinding behavior of mature individuals with DS in a virtual town containing three target buildings and a number of visual landmarks. Participants with DS (CA: 14.2–29.9 years; MA: 7–9 years), and MA-matched and CA-matched TD participants were trained on two different routes, A–B and A–C, consecutively. By the end of training, 10/10 CA, 9/10 MA and 7/10 participants with DS had learned the two routes (evidenced by two consecutive trials without errors). However, participants with DS learned fewer landmarks located along the routes than MA and CA participants. In addition, DS and MA participants made more wrong choices along the routes than CA participants, and the distance traveled by participants with DS on their last trial was longer than that of MA and CA participants. Finally, on a shortcut trial performed by participants who had learned the routes, 10/10 CA participants, 5/9 MA participants and 2/7 DS participants were able to take a previously untraveled shortcut between known routes. Thus, some of these findings suggest specific spatial impairments in individuals with DS, whereas other measures of spatial capacities seem related to MA in both DS and TD individuals.

Similarly, Purser et al. (2014) investigated the development of route learning in DS using virtual environments. They found that both individuals with DS and TD individuals were able to use different types of landmarks (i.e., located near junctions, further from junctions along the route, and distal landmarks) to aid route learning. Nonetheless, in mazes where only junction or route landmarks were available, individuals with DS made more errors than MA-matched TD individuals; low non-verbal ability had a more significant impact on the performance of individuals with DS than on TD individuals. In contrast, in mazes where only distal, extra-maze landmarks were available, although individuals with DS still made more errors than TD individuals, non-verbal ability correlated similarly with performance in both groups of individuals.

### DIFFERENCES BETWEN VIRTUAL AND REAL ENVIRONMENTS

Although virtual environments are often used to assess allocentric capacities in humans (Skelton et al., 2000; Astur et al., 2002; Hamilton et al., 2003), their ethological validity has been questioned (Taube et al., 2013; Banta Lavenex et al., 2014). In the real world, information derived from different sensory modalities is coherent and fully integrated by the brain, including the hippocampus, to elaborate consistent representations of personal experience. In contrast, in virtual reality studies, different inputs derived from different sensory modalities are inconsistent, so that both cooperative and competitive interactions between sensory cues influence hippocampal place cell activity (Ravassard et al., 2013). Accordingly, in the case of a person sitting in front of a computer screen, vestibular, proprioceptive, and tactile information are all coherently coding the absence of movement of the individual, whereas visual information is typically used to make the person believe that s/he is actively or passively moving while exploring the virtual environment. Although one might argue that humans are accustomed to such discrepancies due to their use of modern modes of transportation, one cannot ignore the fact that these conditions are fundamentally different from those experienced in the real world. Indeed, recordings of hippocampal place cells in animals navigating in virtual environments reveal that, as compared to real world navigation, theta frequency is reduced and its speed dependence abolished in rats (Ravassard et al., 2013), and in monkeys fewer place cells are activated and their place fields are smaller (Matsumura et al., 1999). Thus, whereas findings that demonstrate AS competence in virtual environments may be convincing, impaired performance in virtual environments cannot be considered as unequivocal evidence for the impairment of real-world AS capacities, especially in children or individuals with neurodevelopmental disorders or neurological impairments.

### AIM OF THE CURRENT STUDY

In sum, data from two different lines of research lead to two opposing predictions with respect to real-world AS processing capacities in DS. The preserved visuospatial capacities of individuals with DS tested on small-scale spatial tasks predict that individuals with DS should exhibit similarly preserved large-scale, AS capacities. In contrast, the few experimental findings from individuals with DS tested in real-world and virtual tasks designed to assess allocentric capacities suggest that individuals with DS have AS memory impairments. Moreover, reports of specific hippocampal pathology in DS (see Contestabile et al., 2010 for a review) predict that this second hypothesis is more likely. However, additional corroborating evidence is needed to support the hypotheses that (1) DS is associated with its own uneven cognitive profile, reflecting some relatively preserved egocentric visuospatial capacities and impaired AS capacities, and (2) specific hippocampal dysfunction may underlie several aspects of impaired cognition in persons with DS.

In order to better define the nature of AS memory processing in DS, we tested 20 participants with DS and 16 MA-matched TD children in a real-world, AS memory task. Participants were asked to find three rewards hidden among 12 potentially rewarded locations distributed in a 4 m × 4 m arena. On half of the trials, a local cue (LC; a red cup) marked the rewarded locations, thus allowing participants to use a visual guidance strategy to find the rewards. On the other half of the trials, no LC marked the rewarded locations, and instead participants had to form and rely on an allocentric representation of the rewarded locations in order to find the rewards. We hypothesized that participants with DS would perform as well as TD children in presence of LCs, but would be impaired in the AS condition.

## MATERIALS AND METHODS

### PARTICIPANTS AND OVERALL ORGANIZATION OF TESTING

Participants were 20 individuals with DS (10 females, 10 males; average CA: 18.81 years, range: 11.74–29.70 years; average MA: 5.30 years, range: 4.67–6.67 years), and 16 MA-matched, typically developing (TD) children enrolled in the public school system without special education assistance (seven males, nine females; average CA: 4.91 years, range: 4.08–6.07 years; average MA: 4.97 years, range: 3.90–6.16 years). Participants with DS were recruited with the help of the Down Syndrome Family Association (Nardò, Lecce) via the Bambino Gesù Children’s Hospital in Rome which follows individuals with DS for periodical examination. All individuals with DS were diagnosed with free trisomy 21 via karyotyping. Individuals with DS that had neurosensory deficits, such as hypoacusia, serious visual impairment, or epilepsy were not included in the study. All individuals with DS lived with their families. TD participants were recruited via parents in local neighborhoods.

For the AS memory task, participants with DS were tested during two 45-min sessions on two consecutive days. On a separate day, the MA of individuals with DS was evaluated using the Leiter International Performance Scale-Revised (Leiter-R; Subtests included in the Brief IQ from which MA is calculated are: Figure Ground, Form Completion, Sequential Order, and Repeated Patterns; Roid and Miller, 1997). TD participants were tested during two sessions on two consecutive days, one session of ∼45 min and another of ∼30 min. MA was evaluated with the same Leiter-R battery in a separate 30 min session at the end of the second session. All testing took place between 8:00 A.M. and 6:30 P.M. Human subjects research was approved by the Cantonal Ethics Commission (Vaud) for Human Research (protocol no. 60/14), and was in accordance with the NIH guidelines for the use of human subjects in research. The parents of all participants gave informed written consent.

### TESTING FACILITIES

**FIGURE 1:**
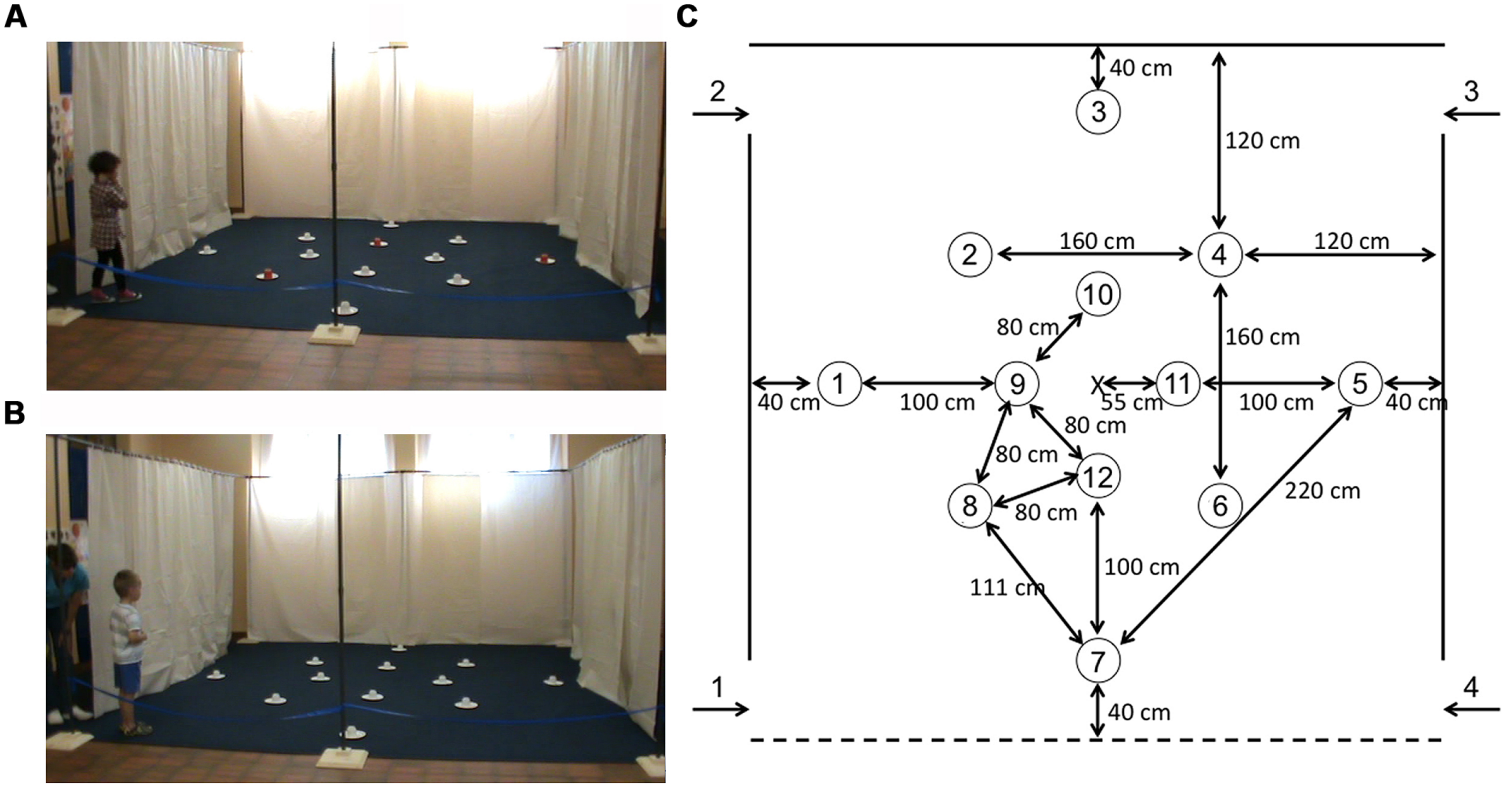
**Experimental setup. (A)** Picture of a TD participant in the arena in the local cue (LC) condition (note the three red cups at positions 5, 8, and 10). **(B)** Picture of a TD participant in the arena in the allocentric spatial (AS) condition (all cups are white). **(C)** Schematic representation of the experimental arena, with the 12 potential locations and their actual positions in the arena. The three rewarded locations were: location 5 on the *outer array*, location 8 on the *middle array*, and location 10 on the *inner array*.

We had testing facilities in two different locations: TD participants were tested in the canton of Vaud, Switzerland (**Figures 1A,B**). Participants with DS were tested in Nardò, Italy. The main features of the testing facilities were consistent between the two sites. Testing took place within large rectangular rooms (9 m × 6 m in Vaud and 12 × 8 m in Nardò) containing many polarizing features such as doors, obscured windows, tables, chairs, wall posters, etc. Within the room, we placed a 4 m × 4 m testing arena (**Figure 1**) that consisted of three walls made of suspended, opaque plastic curtains (2 m high). Whereas the curtain on the back wall was 4 m wide, the curtains on the side walls extended only 3 m, so that there was a 50 cm gap at the front and the back of the wall, thus creating four entry points through which participants passed in order to enter and exit the arena. The fourth (front) boundary of the arena was delineated by a rope attached to the two opposing sides of the arena, and suspended 30 cm off the ground. Exterior to the two side walls, the inter-trial waiting area was a corridor (1 m × 4 m) that contained two chairs with their backs to the arena, and various items including a trash can, occluded windows, doors, posters, etc. Importantly, from within the arena, and from the inter-trial waiting area, participants had access to distant visual cues in front of the arena. Objects found in front of the arena (a table covered with a colorful tablecloth, chairs, the experimenter, camera, etc.) were placed 3 m away from the front of the arena in both the Vaud and Nardò testing rooms.

The arena floors were uniform in both the Vaud and Nardò testing rooms and thus provided no visual guidance cues: In Vaud, a solid blue carpet covered the testing area. In Nardò, the floor consisted of uniform square tiles. The testing arenas were void of all objects except for 12 symmetrically arranged white paper plates (18 cm in diameter; **Figure 1**). The 12 locations were arranged on three nested square arrays (**Figure 1C**): The outer array (rotated 45° with respect to the orientation of the arena) comprised locations 1, 3, 5, 7; The middle array comprised locations 2, 4, 6, 8; The inner array (rotated 45° with respect to the orientation of the arena) comprised locations 9, 10, 11, 12. An inverted opaque plastic cup (7.5 cm in diameter, 6.5 cm high) was placed on each paper plate. A reward was placed under the inverted cups at locations 5, 8, and 10 (**Figure 1**). Participants had to lift or turn over the plastic cups to obtain the reward. Rewards were coins for individuals with DS and “treats” (e.g., Smarties®, Goldfish® crackers, pieces of breakfast cereal or pretzels) for TD children. Parents of TD children were queried with respect to alimentary allergies prior to testing. All testing was videotaped with a video camera located in front of the arena.

### SPECIFIC TESTING PROCEDURES

All testing involved a team of two experimenters. Experimenter 1 (E1) would stay with the participant throughout the testing session and would enter the arena with the participant, encourage the participant to search for the hidden rewards, verbally praise the participant when a reward was found, pick up cups as soon as they had been searched by the participant and place them in a plastic bucket that she carried, direct the participant to the correct exit at the end of the trial, and occupy the participant during the inter-trial interval by reading or talking. Experimenter 2 (E2) was responsible for replacing the rewards between trials, recording the data, and announcing the correct entry and exit doors to E1. Before testing began, participants viewed the arena with the 12 arranged plates (no inverted cups were present), from in front of the arena. While still in front of the arena, E1 then showed the participant a reward item on a paper plate that she held in her hand. While the participant was watching, E1 would lower a plastic white cup over the reward to hide it. The participant would then be asked “Where is the treat/coin? Can you show me where it is?” When the participant lifted the cup to expose the reward, he/she would be verbally praised and told that it was his/hers to keep. Once the participant had been shown that a reward could be found underneath the plastic cup, the participant and E1 would go to the predetermined side of the arena where testing would begin. Once the participant was behind the curtain and occupied, E2 would hide a reward at each of the three predetermined reward locations (locations 5, 8, and 10; **Figure 1**).

Children completed two different types of trials: (1) LC trials, in which a LC, specifically red cups, covered the rewards, whereas all other non-rewarded locations were covered with white cups (**Figure 1**). This condition allowed us to gage participants’ motivation to participate, as well as to test each participant’s ability to find rewards at spatially fixed locations marked by LCs (red cups). In this condition, participants could find and remember the reward locations either by associating the presence of the LC with the reward, or by remembering the absolute spatial locations of the rewards based on their relations to distal environmental objects. (2) AS trials, in which no LCs marked the reward locations, as identical white cups covered all locations. In this case, participants could not discriminate between rewarded and never-rewarded locations based on local features. Instead, participants had to rely on an AS representation of the environment to discriminate these locations, i.e., coding the absolute goal locations in relation to distal environmental objects. Each participant had a total of 20 trials (10 LC and 10 AS trials) distributed over two sessions on two separate days; LC and AS trials alternated. In addition, the first trial of Day 2 was a PT in the AS condition to test participants’ long-term (24 h) memory for the reward locations; the three same locations were rewarded as usual. Following the PT, LC, and AS trials continued in the same alternating manner as for the first day. The entire testing schedule was thus as follows; Day 1: LC_1_, AS_1_, LC_2_, AS_2_, LC_3_, AS_3_, LC_4_, AS_4_, LC_5_, AS_5_; Day 2: PT, LC_6_, AS_6_, LC_7_, AS_7_, LC_8_, AS_8_, LC_9_, AS_9_, LC_10_, AS_10_.

As described above, there were four entries and exits to the arena. Entry order was determined in a pseudo-random manner, with respect to the following conditions: (1) All entrances should be used an equal number of times in the two conditions (LC and AS conditions) across the 2 days; (2) Participants may never enter the arena through a door which they had just exited on the immediately preceding trial (to preclude the use of egocentric strategies); (3) Two successive trials should never have the same entry; and (4) All entries must be made from the same side (right or left) that the participant just exited on the previous trial (i.e., participants were not moved from one side of the arena to the other between trials). At the end of the trial, E2 would call out the appropriate exit number, and E1 would guide the participant to that exit by pointing or by heading there first (all participants were required to walk to and through the exit on their own and were never led by taking their hand), therefore ensuring that the participant was on the appropriate side of the arena for the next trial. Participants were thus constantly moving about the arena from trial to trial, entering and exiting on different sides, and at the back or front of the arena. Moreover, no environmental landmarks, such as doors, windows, or furniture, could be found adjacent to or directly behind any of the reward locations (with the exception of the red cups in the LC condition). Consequently, in order to identify the reward locations in the absence of the LCs, participants must rely on an allocentric, spatial representation of their environment (Banta Lavenex and Lavenex, 2009, 2010; Banta Lavenex et al., 2014).

### VERBAL INSTRUCTIONS AND FEEDBACK

The goal of the experiment was to determine whether each participant possessed the capacity to utilize an allocentric, spatial relational representation of the environment in order to identify the reward locations. However, when working with children or individuals with neurodevelopmental disorders, it is important to ensure that each participant understands the basic requirements to perform the task. Even though we have previously shown that adults need no verbal instructions to exhibit successful performance (Banta Lavenex and Lavenex, 2010), we previously found that children between 48 and 60 months of age are very uncomfortable not receiving any kind of verbal instructions or feedback, and in fact perform worse than younger children (30–45 months) who have fewer problems performing the task without verbal instructions or feedback. Overman et al. (1996) found very similar results in a Morris search task. Thus, in order to give all participants, individuals with DS and MA-matched TD children, the greatest possibility of succeeding, we gave specific verbal instructions and as much feedback/encouragement as possible. Specifically, upon entering the arena on the first trial, E1 would explain to the participant that s/he was going to see some cups, and that if s/he looked under the cups s/he would find some rewards (i.e., “coins” or “treats”). At this point, participants would begin to slowly lift cups one-by-one until they found the rewards. Once a location was searched, E1 would provide verbal feedback, praising the participant for finding a reward, consoling and encouraging the participant when a reward was not found. Verbal instruction/feedback did not vary in quantity or meaning between DS and TD participants. One participant with DS had physical impairments which made it difficult for him to bend over to search under the cups. Instead, this participant would stand next to the cup that he wanted to search and point to it, after which E1 would lift the cup for him.

Although participants were given as much verbal instruction, encouragement, and praise as possible in order to help them perform the task, they were never told or shown where the rewards were, or how to identify their locations. Specifically, they were never told that when the red cups were present they could find the rewards there, nor were they verbally alerted to the spatial relations between distal objects in the room and the reward locations. Both experimenters, and any observing parents, wore dark sunglasses while the participant was in the arena in order to avoid unintentionally cuing the participant as to the locations of the rewards with eye gaze.

Finally, although many participants had a tendency to spontaneously continue to lift other unrewarded cups after they had found the rewards, if they did not, they were encouraged to do so (at least for the first two or three trials) in order to make sure that the participant understood the rules of the game (i.e., which locations hid rewards and which ones did not, and that these locations remained the same from trial to trial). Indeed, in accordance with findings from our previous studies using this task with TD children and adults (Banta Lavenex and Lavenex, 2010; Ribordy et al., 2013), it is critical to let participants explore without penalty as much as they feel necessary, in order to preclude them from exploring before choosing the correct locations, thus confounding their natural desire to explore in order to verify their response and/or the rules of the game with their real performance on the task.

### DATA ANALYSIS

Above chance performance was determined for each individual with a non-parametric Wilcoxon signed-rank test comparing the number of correct choices (visiting a rewarded location) and the number of incorrect choices (visiting a non-rewarded location) for the ten LC trials and the ten AS trials. We considered both the first choice and the first three choices upon entering the arena, and normalized the numbers of choices based on the probability to make those choices: the number of correct choices was divided by three, as there were three rewarded locations, and the number of incorrect choices was divided by nine, as there were nine non-rewarded locations.

Because the latency to solve a task might be influenced by different factors such as confidence, strategy, and motivation, we do not rely on latency as a measure of spatial memory ability. Instead, we determine whether participants are accurate at recalling the reward locations by determining whether and how well they discriminate rewarded locations from non-rewarded locations, thus demonstrating that subjects do or do not remember where the rewards were. The following measures were used to describe and analyze the participants’ behavior and performance: (1) the total number of locations visited to find the three rewards [total number visited (TNV)], an overall measure of task performance; (2) the number of correct locations visited before making an error [correct before error (CBE)], a measure of memory capacity; (3) the number of errorless performers (NEPs) per group for each trial [number errorless performers (NEP)], an evaluation of perfect memory performance; (4) The number of participants who chose a rewarded location as the first location visited upon entering the arena [first choice correct (FCC)]. For these analyses, we performed general linear model (GLM) analyses to compare groups across daily trials. We performed separate analyses for trials in the LC and AS conditions. Because AS tasks requiring low spatial resolution can be solved using either low-resolution topological coding or high-resolution metric coding (Poucet and Benhamou, 1997), and the fact that these two coding mechanisms likely implicate different hippocampal circuits (Lavenex and Banta Lavenex, 2013), we considered the possibility that different locations in the arena might be encoded via different coding strategies and thus remembered differentially. Specifically, whereas topological coding may be used to discriminate location 5 from other non-rewarded locations on the outer array, locations 8 and 10 require precise metric coding in order to be reliably discriminated from surrounding non-rewarded locations. We therefore analyzed the types of locations chosen (either rewarded or non-rewarded) by array (outer, middle, or inner) for (5) the first choice and the first three choices of participants upon entering the arena, averaged over the ten trials in the LC and AS conditions, and (6) for the first choice and the first three choices upon entering the arena during the probe trial (i.e., the first trial of Day 2, in the AS condition). For these analyses, we normalized the number of choices based on the probability to make those choices, by dividing the number of choices of a rewarded location on any array by one and the number of choices of non-rewarded locations on the same array by three. We performed repeated measures GLM analyses to compare groups’ choices. Significance level was set at *p* < 0.05 for all analyses. All statistical analyses were performed with SPSS 18.0 statistical software. There was no sex difference in any of the analyses performed in the study, so we pooled the data from males and females for the presentation of the results.

## RESULTS

### DEMOGRAPHICS, INCLUSION CRITERIA AND AGE CORRELATIONS

**Table 1** presents the demographics of the two groups of participants tested in this study. As planned, there was no difference in MA between individuals with DS and TD children [*t*_(34)_ = 1.444, *p* = 0.158].

**Table 1:** Demographics of the two groups of participants tested in this study.

	Chronological age	IQ	Mental age
**TD (7 M / 9 F)**
Average:	4.91	103	4.97
Stdev:	0.55	11	0.73
Min:	4.08	89	3.90
Max:	6.07	124	6.16
**DS (10 M / 10 F)**
Average:	18.81	41	5.30
Stdev:	5.84	5	0.60
Min:	11.74	36	4.67
Max:	29.70	56	6.67

In order to evaluate each participant’s overall understanding and motivation to perform the task, we determined whether individual participants exhibited selectivity in choosing rewarded locations on the LC trials (Wilcoxon signed-rank test on correct vs incorrect choices). All TD participants (16/16 or 100%) demonstrated a preference for the rewarded locations on the LC trials. Similarly, all but one participant with DS exhibited a preference for the rewarded locations on the LC trials (19/20 or 95%; group comparison: Chi-square = 0.823, *p* = 0.3643). However, two participants with DS (the one that was not selective on LC trials and one other) performed worse than the other participants with DS on the LC trials (more than two SDs from the mean of the DS group in the analyses considered below). Moreover, neither of these two individuals performed at above chance levels on the AS trials (see **Table 2**, below). Thus, since we could not be sure that these two individuals (a 26.3-year-old male with MA = 5.8 years and an 18.7-year-old male with MA = 5.1 years) understood the basic objectives of the task, they were not included in the remaining analyses (unless otherwise noted).

**Table 2:** Numbers of participants who exhibited selectivity for the rewarded locations on either their first or their first three choices upon entering the arena, on LC and AS trials.

	LC trials			AS trials		
	Yes	No		Yes	No	
TD (*n* = 16)	16	0	100%	16	0	100%
DS (*n* = 20)	19	1	95%	10	10	50%
Group comparison	Chi-square: 0.823		*p* = 0.3643	Chi-square: 11.077		*p* = 0.0009

Whereas MA correlated with CA in TD participants (*n* = 16), there was no relation between CA and MA in participants with DS (*n* = 18). Although there was no group difference in MA, we included MA as a covariate in our analyses of TNV, CBE, NEP, and FCC, as we have previously shown a gradual improvement in AS learning and memory capacities with age in TD participants (Ribordy et al., 2013; Ribordy Lambert et al., accepted).

### TOTAL NUMBER OF VISITS (TNV)

**FIGURE 2:**
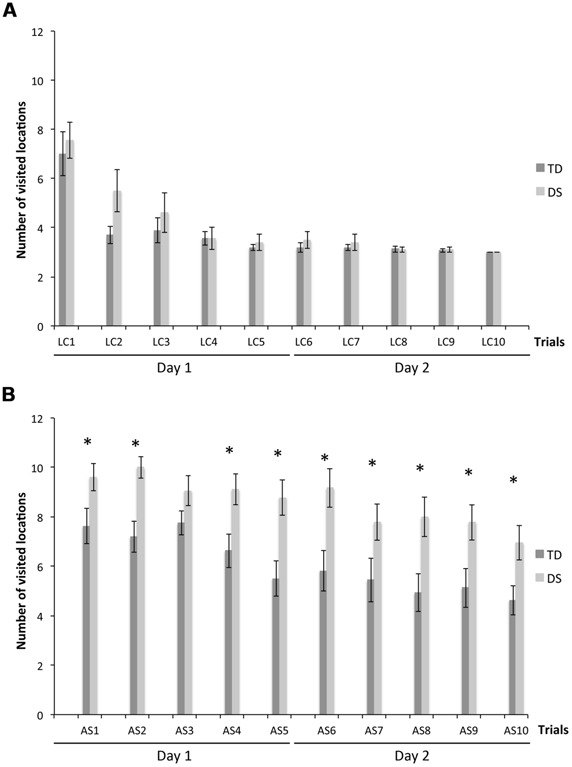
**Total number of visited locations to find the three rewards (TNV). (A)** LC trials. **(B)** AS trials. The asterisks denote statistically significant group differences on a given trial at *p* < 0.05.

We determined the total number of visited locations to find the three rewards, an overall measure of task performance. On LC trials (**Figure 2A**), there was no group [*F*_(1,31)_ = 1.497, *p* = 0.230] or MA effect [*F*_(1,31)_ = 1.090, *p* = 0.304], but a decrease of TNV across trials [*F*_(9,279)_ = 2.184, *p* = 0.023]. TNV decreased from the first to the second trial for TD participants [*F*_(9,135)_ = 13.164, *p* < 0.001; LC_1_ > LC_2_–LC_10_, all *p* < 0.05], and more gradually from the first to the fourth trial for participants with DS [*F*_(9,153)_ = 12.655, *p* < 0.001; LC_1_ > LC_2_ > LC_4_–LC_10_, all *p* < 0.05].

On AS trials (**Figure 2B**), there was an effect of group [*F*_(1,31)_ = 25.855, *p* < 0.001] and MA [*F*_(1,31)_ = 12.701, *p* < 0.001], and a non-significant decrease of TNV across trials [*F*_(9,279)_ = 1.386, *p* = 0.194]. However, considering each group separately, TNV decreased gradually across trials for both TD [*F*_(9,135)_ = 5.385, *p* < 0.001] and DS [*F*_(9,153)_ = 3.134, *p* = 0.002] participants. TNV was lower for TD participants than DS participants on all AS trials, except for AS_3_ (all *p* < 0.05).

### NUMBER OF CORRECT CHOICES BEFORE ERRING (CBE)

**FIGURE 3:**
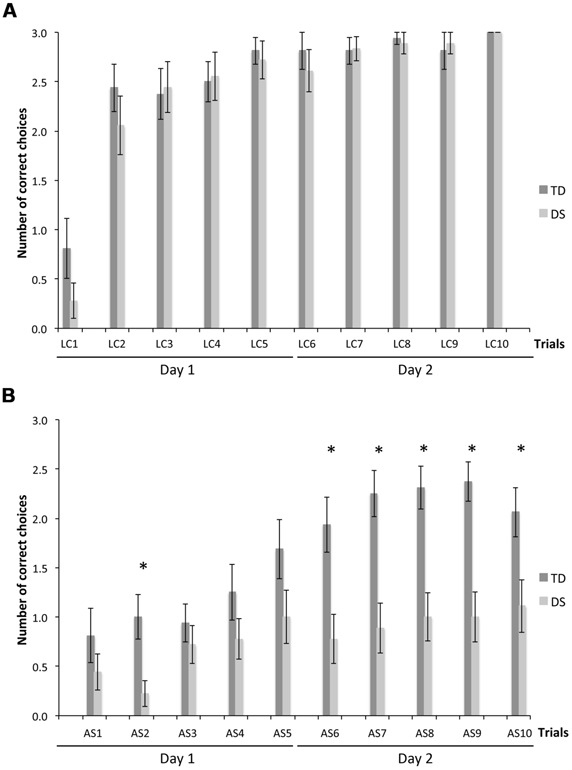
**Number of correct choices before erring (CBE), a measure of memory capacity. (A)** LC trials. **(B)** AS trials. The asterisks denote statistically significant group differences on a given trial at *p* < 0.05.

We determined the number of correct choices participants made before making an error, i.e., visiting a non-rewarded location, a measure of memory capacity. On LC trials (**Figure 3A**), there was no group [*F*_(1,31)_ = 0.830, *p* = 0.369] or MA effect [*F*_(1,31)_ = 1.330, *p* = 0.258]. CBE increased significantly from the first to the second trial for TD participants [*F*_(9,135)_ = 13.047, *p* < 0.001; LC_1_ < LC_2_–LC_10_, *p* < 0.05] and more gradually from the first to the fourth trial for participants with DS [*F*_(9,153)_ = 26.548, *p* < 0.001; LC_1_ < LC_2_ < LC_4_–LC_10_, *p* < 0.05].

On AS trials (**Figure 3B**), there was an effect of group [*F*_(1,31)_ = 28.792, *p* < 0.001] and MA [*F*_(1,31)_ = 21.506, *p* < 0.001], and an interaction between groups and trials [*F*_(9,279)_ = 3.462, *p* < 0.001]. Considering each group separately, CBE increased gradually across trials for both TD participants [*F*_(9,135)_ = 12.574, *p* < 0.001] and participants with DS [*F*_(9,153)_ = 2.946, *p* = 0.003]. CBE was higher for TD participants than participants with DS in the second and the last five AS trials (AS_2_, AS_6_–AS_10_; all *p* < 0.05).

### NUMBER OF ERRORLESS PERFORMERS

**FIGURE 4:**
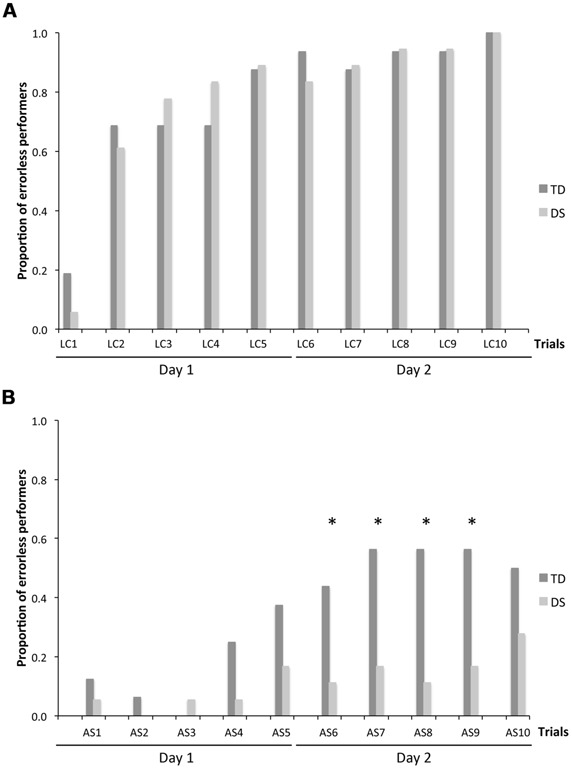
**Number of errorless performers (NEP), expressed as a proportion of participants in each group: *n***=** 16 for TD participants and *n***=** 18 for participants with DS. (A)** LC trials. **(B)** AS trials. The asterisks denote statistically significant group differences on a given trial at *p* < 0.05.

Because CBE is expressed as an average number of locations remembered across individuals, it does not give an indication as to whether some participants exhibited perfect memory performance on some trials. We thus determined, for each trial, the number of participants per group who made no errors. On LC trials (**Figure 4A**), there was no group [*F*_(1,31)_ = 0.116, *p* = 0.736] or MA effect [*F*_(1,31)_ = 1.592, *p* = 0.216]. NEP tripled from the first to the second trial, and increased gradually after the second trial to include all participants on the tenth LC trial, for both the TD group [F_(9,135)_ = 9.542, *p* < 0.001; LC_1_ < LC_2_–LC_10_, all *p* < 0.05] and the group with DS [*F*_(9,153)_ = 20.828, *p* < 0.001; LC_1_ < LC_2_–LC_10_, all *p* < 0.05].

On AS trials (**Figure 4B**), there was an effect of group [*F*_(1,31)_ = 18.949, *p* < 0.001] and MA [*F*_(1,31)_ = 24.003, *p* < 0.001], as well as an increase in NEP with trials [*F*_(9,279)_ = 3.830, *p* < 0.001], and significant interactions between groups and trials [*F*_(9,279)_ = 3.462, *p* < 0.001], and between MA and trials [*F*_(9,279)_ = 3.462, *p* < 0.001]. Considering each group separately, NEP increased gradually across trials for both the TD group [*F*_(9,135)_ = 6.643, *p* < 0.001] and the group with DS (*F*_(9,153)_ = 1.972, *p* = 0.046]. NEP was higher for the TD group than for the DS group in trials AS_6_–AS_9_ (all *p* < 0.05).

### NUMBER OF PARTICIPANTS WITH THE FIRST CHOICE CORRECT

**FIGURE 5:**
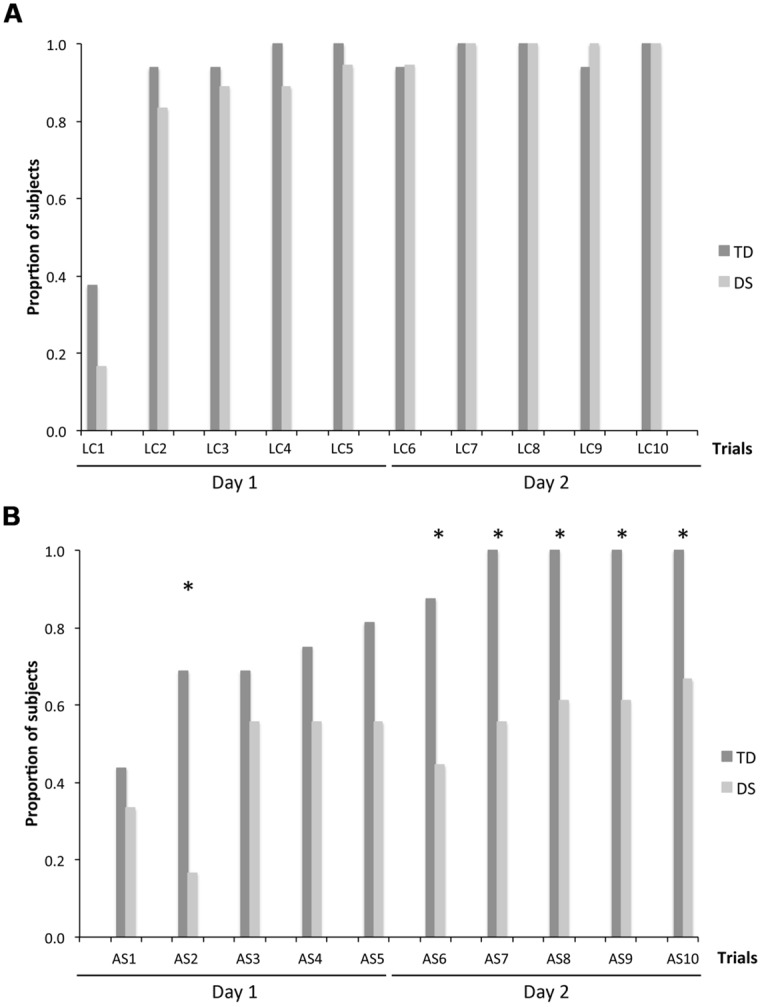
**Number of participants whose first choice was correct (FCC), expressed as a proportion of participants in each group: *n***=** 16 for TD participants and *n***=** 18 for participants with DS. (A)** LC trials. **(B)** AS trials. The asterisks denote significant group differences on a given trial at *p* < 0.05.

We also analyzed the number of participants who chose a rewarded location as their first choice upon entering the arena, in order to determine whether participants with DS exhibited memory capacities that would not be revealed by the more stringent measures of performance described above. On LC trials (**Figure 5A**), there was no group [*F*_(1,31)_ = 2.431, *p* = 0.129] or MA effect [*F*_(1,31)_ = 0.567, *p* = 0.457]. The number of participants with the FCC increased drastically from the first to the second trial and remained stable thereafter, including about 90% of participants for both the TD group [*F*_(9,135)_ = 9.542, *p* < 0.001; LC_1_ < LC_2_–LC_10_, all *p* < 0.05] and the DS group [F_(9,153)_ = 19.905, *p* < 0.001; LC_1_ < LC_2_–LC_10_, all *p* < 0.05].

On AS trials (**Figure 5B**), there was an effect of group [*F*_(1,31_) = 21.930, *p* < 0.001] and MA [*F*_(1,31)_ = 5.894, *p* = 0.021], as well as an increase in the number of participants with the FCC with trials [*F*_(9,279)_ = 2.658, *p* = 0.006], and an interaction between MA and trials [*F*_(9,279)_ = 1.926, *p* = 0.048]. Considering each group separately, FCC increased gradually across trials for both the TD group [*F*_(9,135)_ = 6.024, *p* < 0.001] and the DS group [*F*_(9,153)_ = 2.195, *p* = 0.025]. FCC was higher for the TD group than the group with DS in the second and the last five AS trials (AS_2_, AS_6_–AS_10_; all *p* < 0.05).

### INDIVIDUAL PERFORMANCE IN AS TRIALS

Although group analyses revealed clear differences between DS and TD groups on AS trials (**Figures 2–5**), individual performance seemed to vary greatly among participants with DS. Thus, in order to evaluate each participant’s allocentric capacities, we determined whether individual participants choose rewarded locations on the AS trials significantly more often than non-rewarded locations (**Table 2**; includes all 20 participants with DS originally included in the study). Above chance performance was determined for each individual with a non-parametric Wilcoxon signed-rank test comparing the number of correct choices (visiting a rewarded location) and the number of incorrect choices (visiting a non-rewarded location) for the first and the first three choices made during the ten AS trials. As described above, all TD participants, and all but one participant with DS, exhibited a preference for the rewarded locations on LC trial. In contrast, for the AS trials, whereas all TD participants demonstrated an above chance level preference for the rewarded locations, only 50% of participants with DS did so.

**FIGURE 6:**
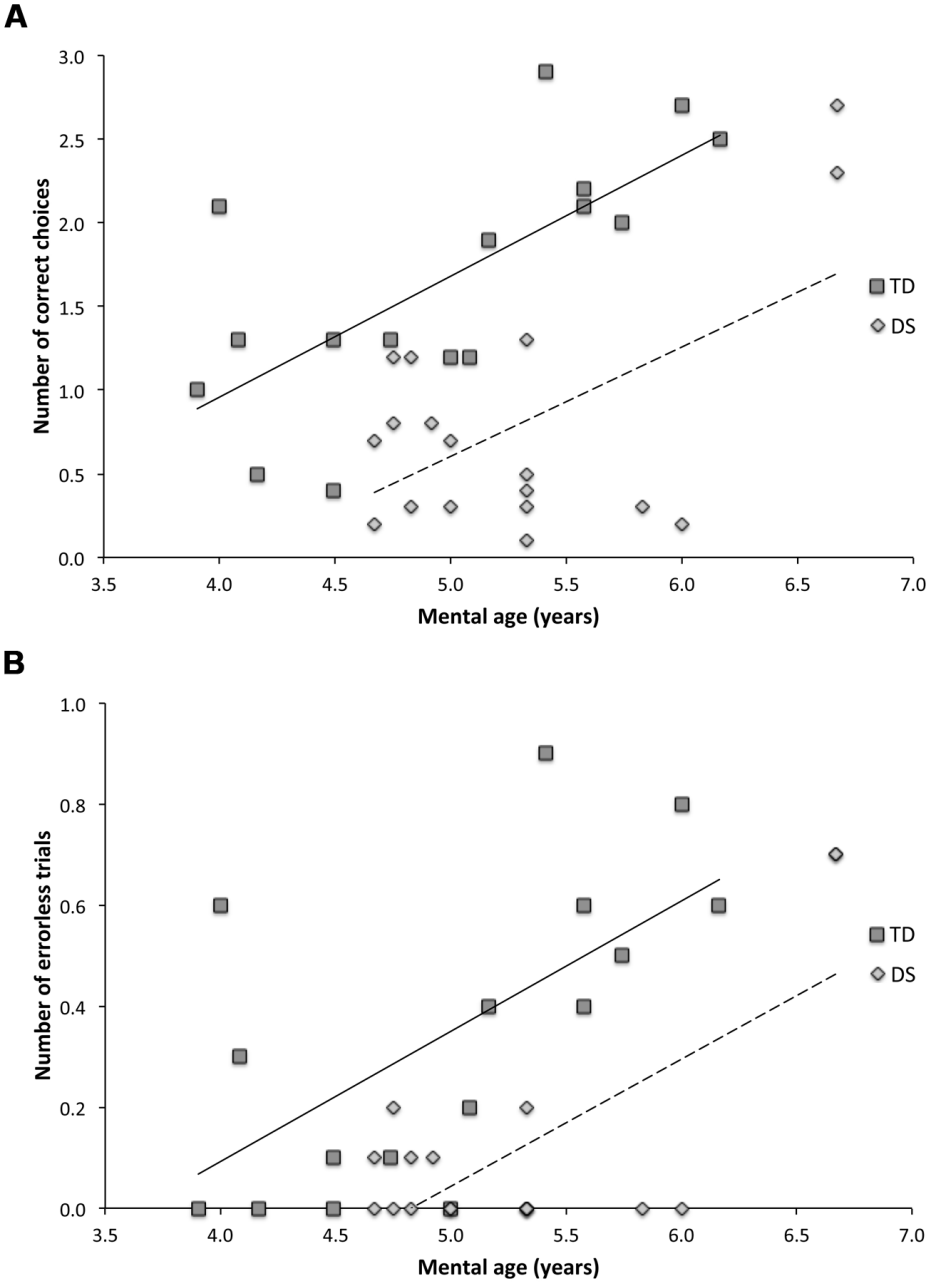
**Relationship between mental age (MA) and task performance in TD children and participants with DS. (A)** Number of correct choices before erring (CBE) in the AS condition. Typically developing (TD) participants : CBE = 0.719 × MA – 1.920, *R*^2^ = 0.502, *F*_(1,14)_ = 14.090, *p* = 0.002. Participants with Down syndrome (DS): CBE = 0.676 × MA – 2.779, *R*^2^ = 0.344, *F*_(1,16)_ = 8.398, *p* = 0.010. The slopes did not differ [*t*_(30)_ = 0.226, *p* = 0.8224]. **(B)** Number of errorless trials (NET) in the AS condition. TD participants: NET = 0.257 × MA – 0.937, *R*^2^ = 0.394, *F*_(1,14)_ = 9.098, *p* = 0.009. Participants with DS: NET = 0.258 × MA – 1.248, *R*^2^ = 0.50184, *F*_(1,16)_ = 17.990, *p* = 0.001. The slopes did not differ [*t*_(30)_ = 0.059, *p* = 0.9526].

We therefore analyzed the individual performance of participants across the ten AS trials based on the number of correct choices before erring (CBE; as a proxy to estimate memory capacity; **Figure 6A**) and the number of errorless trials (NET, as a measure of perfect memory performance; **Figure 6B**). These two analyses confirmed the MA effects reported previously via the GLM analyses of daily trials, and further revealed that, despite significant group differences, task performance improved similarly with MA for both TD children and participants with DS. Note, however, that the MA effect observed in participants with DS appeared largely due to the performance of the two participants with MAs of 6.7 years.

### LOCATION CHOICES IN AS TRIALS

Because AS tasks requiring low spatial resolution can be solved using either allocentric topological coding or precision metric coding (Poucet and Benhamou, 1997), and the fact that these two coding mechanisms likely implicate different hippocampal circuits (Lavenex and Banta Lavenex, 2013), we considered the possibility that different locations in the arena might be encoded via different coding strategies and thus remembered differentially. Specifically, whereas topological coding may be used to discriminate location 5 from other non-rewarded locations on the outer array, locations 8 and 10 require precise metric coding in order to be reliably discriminated from surrounding non-rewarded locations. Thus, in order to further characterize the group differences that had been revealed by our various measures of task performance, we analyzed the types of locations participants visited first upon entering the arena, across the ten AS trials. Since ten participants with DS exhibited selectivity for the rewarded locations on AS trials and eight did not (**Table 2**), we included two DS sub-groups (DSyes, *n* = 10 and DSno, *n* = 8; note that the two participants with DS who exhibited poor performance in the LC trials are not included in these analyses) and the TD group in these analyses.

**FIGURE 7:**
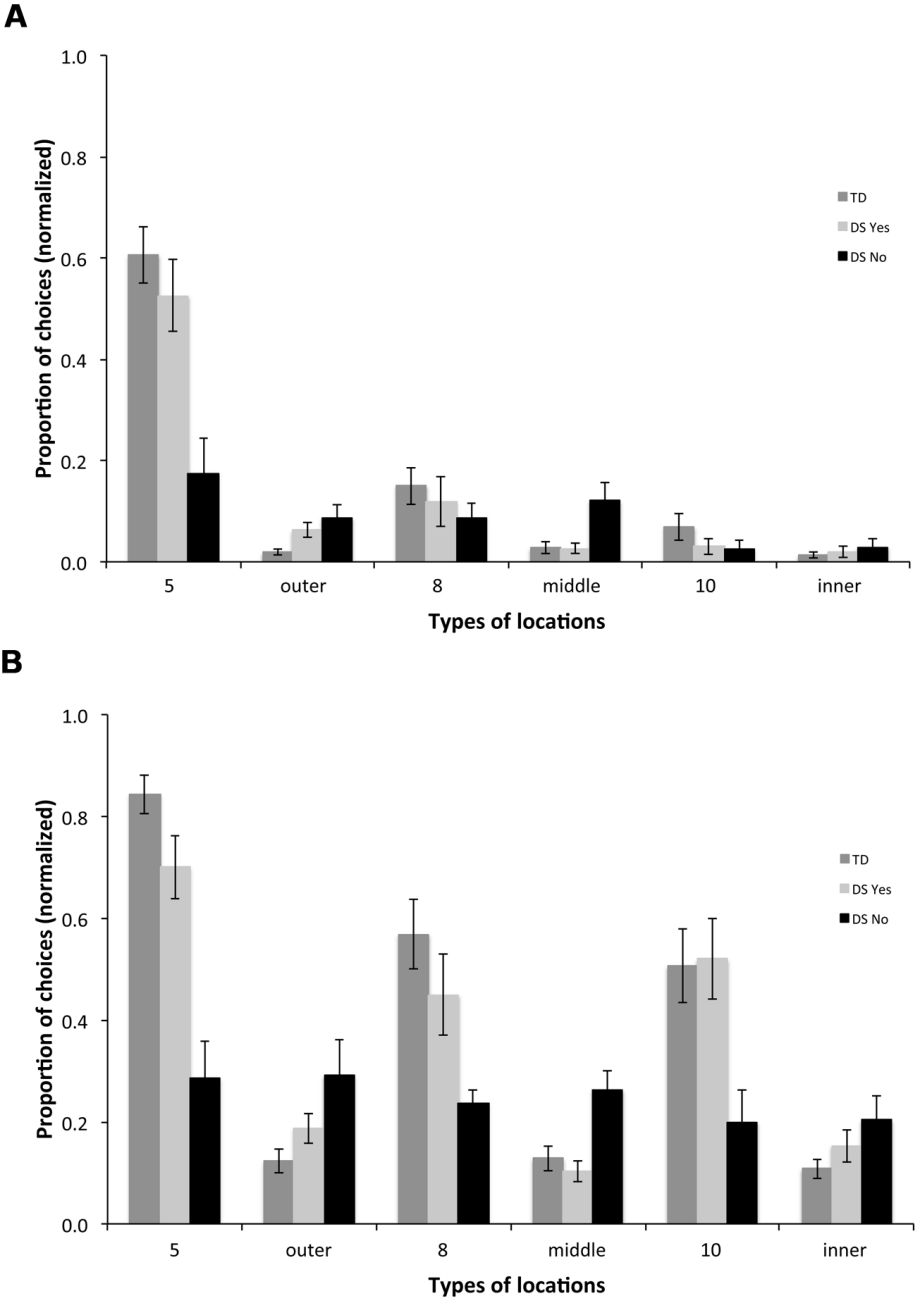
**Types of locations visited for the first **(A)** and the first three **(B)** choices upon entering the arena in the AS condition.** 5: rewarded location on the outer array; outer: non-rewarded locations on the outer array; 8: rewarded location on the middle array; middle: non-rewarded locations on the middle array; 10: rewarded location on the inner array; inner: non-rewarded locations on the inner array.

For the first choice upon entering the arena, we found a group [*F*_(2,31)_ = 21.219, *p* = < 0.001] and a choice effect [*F*_(5,155)_ = 50.063, *p* < 0.001], as well as an interaction between groups and choices [*F*_(10,155)_ = 6.634, *p* < 0.001]. TD participants chose the rewarded location on the outer array (location 5) more than any other location [**Figure 7A**; *F*_(5,75)_ = 51.211, *p* < 0.001; all *p* < 0.05], the rewarded location on the middle array (location 8) more than non-rewarded middle locations (*p* < 0.05), and the rewarded location on the inner array (location 10) more than non-rewarded inner locations (*p* < 0.05). DSyes participants chose rewarded location 5 on the outer array more than any other location [*F*_(5,45)_ = 25.277, *p* < 0.001; all *p* < 0.05]; they did not discriminate other rewarded locations from non-rewarded locations on the middle and inner arrays for their first choice. DSno participants did not discriminate any of the different types of locations [*F*_(5,35)_ = 1.956, *p* = 0.110].

For the first three choices upon entering the arena, we found a group [**Figure 7B**; *F*_(2,31)_ = 16.046, *p* = < 0.001] and a choice effect [*F*_(5,155)_ = 30.850, *p* < 0.001], and an interaction between groups and choices [*F*_(10,155)_ = 7.717, *p* < 0.001]. TD participants chose the outer rewarded location 5 more than any other location [*F*_(5,75)_ = 41.609, *p* < 0.001; all *p* < 0.05], the middle rewarded location 8 more than non-rewarded middle locations (*p* < 0.05), and the inner rewarded location 10 more than non-rewarded inner locations (*p* < 0.05). DSyes participants also chose the outer rewarded location 5 more than any other location [*F*_(5,45)_ = 17.220, *p* < 0.001; all *p* < 0.05, except for 5 vs. 10, *p* = 0.068], the middle rewarded location 8 more than non-rewarded middle locations (*p* < 0.05), and the inner rewarded location 10 more than non-rewarded inner locations (*p* < 0.05). DSno participants did not discriminate between any of the different types of locations [*F*_(5,35)_ = 0.437, *p* = 0.819].

### TYPES OF CHOICES IN THE PROBE TRIAL

In order to evaluate the participants’ long-term (24 h) memory, we analyzed the types of locations participants visited first upon entering the arena on a probe trial, the first trial on Day 2 performed in the AS condition. We compared the TD group and the two sub-groups with DS (DSyes, *n* = 10 and DSno, *n* = 8).

**FIGURE 8:**
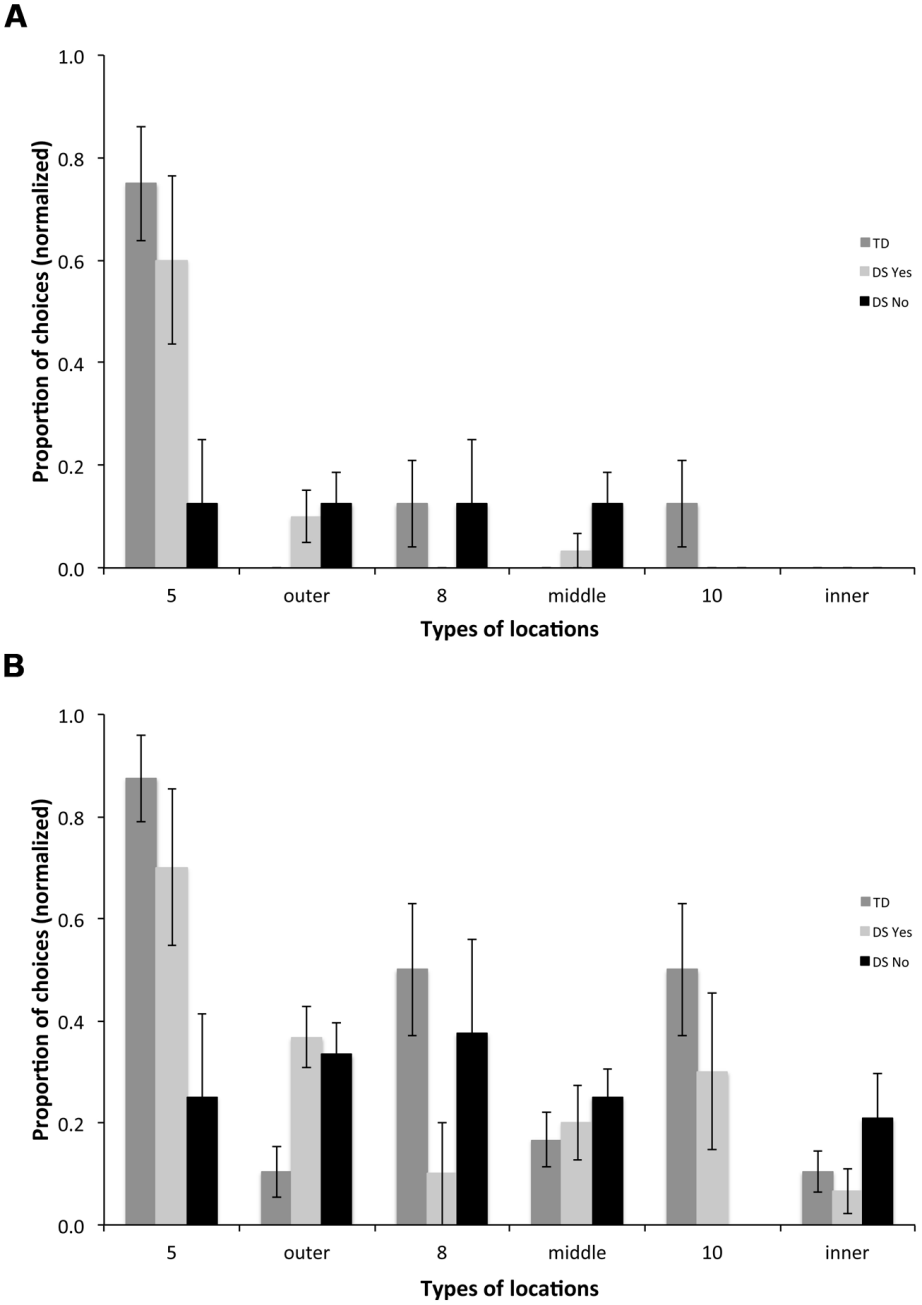
**Types of locations visited for the first **(A)** and the first three **(B)** choices upon entering the arena in the probe trial, the first trial of Day 2 performed in the AS condition.** 5: rewarded location on the outer array; outer: non-rewarded locations on the outer array; 8: rewarded location on the middle array; middle: non-rewarded locations on the middle array; 10: rewarded location on the inner array; inner: non-rewarded locations on the inner array.

For the first choice upon entering the arena, we found a group [*F*_(2,31)_ = 12.554, *p* < 0.001] and a choice effect [*F*_(5,155)_ = 14.575, *p* < 0.001], and an interaction between groups and choices [*F*_(10,155)_ = 3.215, *p* < 0.001]. TD participants chose the outer rewarded location 5 more than any other location [**Figure 8A**; *F*_(5,75)_ = 15.769, *p* < 0.001; all *p* < 0.05]; they did not discriminate the other rewarded locations 8 and 10 from non-rewarded locations. DSyes participants also chose the outer rewarded location 5 more than any other location [*F*_(5,45)_ = 9.949, *p* < 0.001; all *p* < 0.05]; they did not discriminate the other rewarded locations 8 and 10 from non-rewarded locations. DSno participants did not discriminate any of the different types of locations [*F*_(5,35)_ = 0.565, *p* = 0.726].

For the first three choices upon entering the arena, we found a group [*F*_(2,31)_ = 6.370, *p* = 0.005] and a choice effect [*F*_(5,155)_ = 6.675, *p* < 0.001], and an interaction between groups and choices [*F*_(10,155)_ = 3.397, *p* < 0.001]. TD participants chose the outer rewarded location 5 more than any other location [**Figure 8B**; *F*_(5,75)_ = 10.941, *p* < 0.001; all *p* < 0.05], the middle rewarded location 8 more than non-rewarded middle locations (*p* = 0.063), and the inner rewarded location 10 more than non-rewarded inner locations (*p* = 0.018). DSyes participants chose the outer rewarded location 5 more than non-rewarded locations [*F*_(5,45)_ = 4.329, *p* = 0.003; 5 vs. outer, *p* = 0.095; 5 vs. middle, *p* = 0.030; 5 vs. inner, *p* = 0.004]; they did not discriminate other rewarded locations from non-rewarded locations. DSno participants did not discriminate any of the different types of locations [*F*_(5,35)_ = 1.177, *p* = 0.340].

## DISCUSSION

### ALLOCENTRIC SPATIAL MEMORY DEFICITS IN DS

In the current study, we found that individuals with DS were able to discriminate the rewarded locations in presence of LCs as well as MA-matched TD children. This finding confirms that participants with DS understood the basic objectives of the task, and that they could initiate and sustain a selective search, including inhibiting searching unrewarded locations, when they knew where the rewards were hidden. It also confirms that the fact that the two groups of participants were tested in two different locations, and given instructions in their native language (in Italian by a bilingual experimenter (GK) for the DS participants in Nardò, and in French for the TD children in Vaud), did not impact the reported findings.

In contrast, as a group, individuals with DS discriminated the rewarded locations on AS trials, in absence of LCs, significantly less well than MA-matched TD children. Participants with DS made fewer correct choices before erring, visited more locations in order to find the three rewards, had fewer errorless performers, and fewer correct first choices than MA-matched TD children. It is important to recognize that errors in our task, i.e., visiting unrewarded locations, could not be due to perseverative (working memory) errors since once a location was searched, the cup associated with that location was removed. Thus, incorrect searches represent spatial memory errors, signifying that participants either (1) mistakenly believed that the searched location hid a reward, or (2) did not know where the rewards were hidden.

However, analysis of individual performance revealed that not all individuals with DS performed the same. Our results indicate that for 50% of the participants with DS (10/20) the ability to solve a complex AS memory task with multiple goal locations distributed amongst decoy locations was beyond their capacity. In contrast, 50% of the participants with DS (10/20) were capable of discriminating rewarded locations on AS trials at above chance level. Among those, two individuals with DS (MA > 6.5 years) consistently performed errorless AS trials, and had scores similar to MA-matched TD children.

Thus, in order to summarize our results, we must choose between two views. On the one hand, our finding that 50% of participants with DS performed above chance on AS trials leads to seeing “the glass half full.” On the other hand, our finding that 50% of participants with DS did not perform above chance on AS trial leads to seeing “the glass half empty.” However, it must be kept in mind that even though 50% of the individuals with DS exhibited above-chance performance, for the majority of these individuals (8/10), their performance was still impaired compared to that of MA-matched TD controls. Moreover, even though 48-month-old TD children exhibited above-chance performance on our task, AS memory capacities are still improving at this age and have not reached adult-like levels, or even the level that they will achieve at 6.5 years (**Figure 6**). Thus, considering all of our findings, it seems most accurate and parsimonious to conclude that AS learning and memory capacities are significantly impaired in DS.

### SPATIAL MEMORY PERFORMANCE AND MENTAL AGE

We, along with others, have previously shown that AS memory processes emerge around 2 years of age in TD children (Newcombe et al., 1998; Ribordy et al., 2013), and that AS resolution improves from 2 to 4 years of age (Ribordy et al., 2013; Ribordy Lambert et al., accepted). The present study further reveals that AS memory processing continues to improve beyond 4 years of age, showing a positive correlation between spatial memory performance and MA in TD children between 4 and 7 years (**Figure 6**). We also found a positive correlation between performance and MA in participants with DS. However, it appears that this correlation was strongly influenced by the above-average performance of two participants with DS. It is of particular interest to note that the two participants with DS that performed similarly to MA-matched TD children had MAs of 6.7 years, and that this was the highest MA of all of our DS participants.

A number of previous studies have suggested that AS competence improves with age in TD children, and that mature, adult-like competence is not achieved until around 7 years of age [see (Newcombe and Huttenlocher, 2000) for detailed discussion]. For participants with DS and a MA of less than 6 years, there is no correlation between MA and performance on our task, whereas for TD children, in contrast, the correlation between MA and performance is evident even if we consider only those children younger than 6 years. Because proper hippocampal function is critical for AS learning and memory (Banta Lavenex et al., 2014), and AS memory performance correlates with MA (Ribordy et al., 2013; Ribordy Lambert et al., accepted, current study), it is reasonable to ask whether there is a causal relationship between hippocampal development and MA in typical development. Specifically, does proper hippocampal function influence cognitive performance in general? Moreover, can variations in spatial memory capacities in DS be informative about brain functions that underlie the cognitive processes contributing to the definition of MA in DS. Do individual variations in discrete hippocampal (dys)functions affect some of the cognitive processes that are typically assessed, and define MA, in standard neuropsychological evaluations?

### EVIDENCE OF HIPPOCAMPAL DYSFUNCTION IN DS

It has been previously suggested that DS is characterized as a syndrome impacting late-developing brain structures such as the hippocampus, the prefrontal cortex and the cerebellum (Uecker et al., 1993; Pennington et al., 2003; Menghini et al., 2011; Edgin, 2013). Given the numerous neuroanatomical abnormalities that have been noted in the fetal DS brain, due in large part to widespread reduction in the proliferation of neuronal precursors (Contestabile et al., 2010; Guidi et al., 2011), it seems unlikely that cognitive deficits in DS would be limited to impairments arising from deficient hippocampal or prefrontal processing. Nevertheless, due to the important role that the hippocampus plays in learning and memory, it is of particular interest to determine whether, and to what extent, hippocampus-dependent memory functions are impaired in DS.

#### Spatial memory

Five prior studies have investigated the spatial capacities of individuals with DS. Mangan (1992) tested very young DS and TD children (16–28 months) on three different tasks designed to assess spatial and non-spatial capacities. Two of these tasks, a cue learning task and an egocentric response learning task, are hippocampus-independent tasks requiring dorsal striatal and parietal cortex involvement (White and McDonald, 2002; Weniger et al., 2009). In contrast, the third task, a place learning task, requires hippocampus involvement. Although children with DS were capable of learning to solve all three tasks, children with DS were more greatly impaired on the hippocampus-dependent AS task than on the hippocampus-independent spatial tasks. Pennington et al. (2003) compared the performance of DS and MA-matched TD children on an extensive series of tests evaluating independent behavior (Scales of Independent Behavior), general cognition [Differential Abilities Scale (DAS), PPVT, CANTAB Spatial Span, TROG, CELF, DAS Recall of Digits], hippocampal function, and prefrontal function. They found deficits on all tasks believed to depend on hippocampal function, including a virtual Morris water maze task. It is important to note, however, that Edgin et al. (2010) did not find significant differences between individuals with DS and MA-matched TD children on a similar virtual Morris water maze task, potentially highlighting the relatively poor performance of young MA-matched TD children on virtual spatial tasks. Courbois et al. (2013) investigated DS wayfinding abilities in a virtual environment. Although participants with DS could learn the required routes through the virtual environment, they needed more trials to reach criterion than participants of the same CA, and fewer DS than CA participants, were able to make a shortcut in the virtual environment (2 of 7 vs. 10 of 10), demonstrating impairments in their ability to use allocentric representations in virtual environments. Similar to the Edgin et al., (2010) study, however, Courbois et al., also found that only 5 of 9 MA-matched TD children were able to make the shortcut in the virtual environment. In further virtual route-learing experiments, Purser et al. (2014) found that individuals with DS made more errors than MA-matched TD children using proximal cues (junction and path landmarks) within the maze, that errors were associated with poor inhibition and overall cognitive ability, and that individuals with DS with low non-verbal ability were more impaired than individuals with higher non-verbal ability. In contrast, when participants were required to use distal visual landmarks found outside the maze, they found that the developmental trajectory of performance (i.e., the relationship between non-verbal abilities and the number of errors in the virutal maze) did not differ between individuals with DS and MA-matched TD individuals. In sum, whereas all of these studies demonstrate that individuals with DS exhibit general impairments in spatial learning, the studies conducted in virtual envrionments also show that young MA-matched TD children have difficulty with the purportedly more allocentric aspects of the task, and that the performance of individuals with DS and MA-matched TD children do not necessarily differ on these aspects.

In contrast, in our study, although 50% of the participants with DS demonstrated above chance performance in our AS task, all MA-matched TD children demonstrated above chance performance. Futhermore, the performance of 90% of the individuals with DS we tested was below that of MA-matched TD children. Thus, considering the previous suggestive but not unequivocal findings cited above, together with our current findings showing significant impairments in real-world AS memory processing in 90% of individuals with DS, it seems reasonable to conclude that hippocampus-dependent spatial learning and memory are consistently impaired in DS, and that individuals with DS exhibit performance consistently below that of MA-matched TD controls.

#### Explicit memory

Explicit memory, our ability to bring to mind or recall a stored memory, has been shown to be dependent on hippocampal integrity, whereas implicit memory has been shown to be essentially preserved following hippocampal damage (Squire, 1992). Carlesimo, Vicari and colleagues therefore reasoned that if the hippocampal memory system was disproportionately impaired, participants with DS should perform relatively better on implicit memory tasks, and relatively worse on explicit memory tasks. Carlesimo et al. (Carlesimo et al., 1997) and Vicari et al. (Vicari et al., 2000) compared the performance of individuals with DS on tests of implicit memory (e.g., Tower of London task; Fragmented Picutres task; Serial Reaction Time test; and Stem Completion test), and explicit memory (e.g., Free Recall of a list of unrelated words; explicit recognition of previously viewed words; Corsi Supraspan test; and explicit recognition of previously viewed images; prose recall). Both studies showed that whereas participants with DS exhibited normal implicit memory capacities, and especially MA-appropriate levels of priming, their explicit memory capacities were impaired as compared to TD children. Thus, similar to AS memory studies, explicit memory studies also suggest that the hippocampus-dependent memory system is specifically impaired in DS.

#### Contextual memory

Context, the visual, spatial or situational details associated with a memory, can facilitate memory recall (Lever and Burgess, 2012). Because the hippocampal formation is known to integrate and associate (bind together) multiple components of perceived objects, locations and events, it is believed to play a prominent role in contextual processing (Hoscheidt et al., 2010; Ognjanovski et al., 2014; Pfeiffer et al., 2014). Edgin et al. (2014) studied the effect of context on object recognition in individuals with DS (10–29 years) and TD children (3–6 years) and adolescents (10–16 years). Interestingly, they found that the performance of participants with DS was similar to that of the 3–4.5 year old children in that they exhibited better recognition when test objects were viewed in the same context in which they had been seen initially than when their context was absent. This type of performance is thought to correspond to a nascent stage of processing that is driven by the encoding and recall of unitized (fused) object and context representations. Indeed, when viewed out of context, 3–4.5 year old TD children and individuals with DS do not judge previously seen objects as familiar. These results thus suggest that hippocampal processing in adolescents and adults with DS (10–29 years old) is similar to that of 3–4.5 year old TD children. Interestingly, our results are in agreement with this estimation. In our task, the majority of participants with DS performed similarly to our poorest performing TD participants that were between 4 and 4.5 years of age.

In sum, studies examining a range of different types of hippocampus-dependent memory, from the explicit recall of verbal, visual and visuospatial material (Vicari et al., 2000; Carlesimo et al., 2001; Pennington et al., 2003), to contextual binding (Edgin et al., 2014), to AS memory capacities (Pennington et al., 2003; Courbois et al., 2013; current study), have demonstrated significant impairments in DS. The fundamental role of the hippocampus in learning and memory for explicit, autobiographical, relational, and AS information, as well as its role in both short-term and long-term memory (Scoville and Milner, 1957; Squire, 1992; Eichenbaum and Cohen, 2001; Ranganath and Blumenfeld, 2005; Hoscheidt et al., 2010; Hunsaker and Kesner, 2013; Banta Lavenex et al., 2014) likely mean that global impairments in hippocampal function, as would be seen following structure-wide reductions in cell proliferation (Contestabile et al., 2010; Guidi et al., 2011), will end up further affecting other aspects of cognition. The high degree of interconnection between the hippocampus and other cortical regions could mean that disrupted hippocampal processing might act synergistically with inefficient processing in other cortical areas to produce greater deficits than might be expected based on processing deficits in individual cortical structures alone. Future studies should try to asses the contribution that hippocampal impairment might have on both general aspects of cognition, such as the relation between hippocampal function and MA, as well as specific aspects of cognition such as short-term memory for non-spatial information (Banta Lavenex et al., 2014).

### DISRUPTION OF DISTINCT HIPPOCAMPAL CIRCUITS IN DS

The hippocampal formation has been proposed to subserve two complementary but partially dissociable spatial coordinate systems (Poucet and Benhamou, 1997). The CA1 region of the hippocampus is thought to subserve allocentric topological coding, where locations are coded in relation to distal environmental objects in a relatively gross manner (location X is closer to the window than it is to the door), which does not necessitate high-resolution spatial representation. In contrast, the dentate gyrus and its projection to CA3 is thought to subserve high-resolution metric coding of space and the process of spatial pattern separation, which serves to transform neural representations of locations into more dissimilar, non-overlapping neural representations (Gilbert et al., 1998). Support for this bipartite system comes from experiments showing that lesions of the dentate gyrus do not entirely disrupt AS memory capacities, as rodents are still able to find one goal location in the Morris water maze (Brun et al., 2002; Nakashiba et al., 2008), but they do disrupt the animals’ ability to distinguish closely apposed locations in AS memory tasks (Gilbert et al., 1998, 2001; Gilbert and Kesner, 2006).

Knowledge of the different computational roles played by distinct hippocampal circuits offers the possibility of using paradigms that can distinguish between behavioral deficits potentially due to pathology in distinct regions of the hippocampal formation. For example, if participants with DS demonstrate that they can learn and remember locations that can be discriminated using allocentric topological coding (location 5 in the current study), but not locations which require high-resolution spatial encoding (locations 8 and 10), this would suggest that the CA1 region of the hippocampus and its afferent circuitry might be relatively intact, but that the dentate gyrus/CA3 region is relatively impaired. In contrast, if participants with DS are incapable of learning to discriminate even locations that, theoretically, can be discriminated using allocentric topographical coding (location 5), this would suggest that CA1, the dentate gyrus and CA3 are similarly impaired.

In the current study, we saw limited behavioral evidence potentially reflecting selective impairment in distinct hippocampal circuits in DS. During the regular AS trials and the probe trial, TD children showed consistent evidence of being able to discriminate all rewarded locations (locations, 5, 8, 10) from never-rewarded locations, suggesting both topological and high-resolution coding capacities. In contrast, the 50% of participants with DS who exhibited below chance performance on AS trials did not show any evidence of being able to discriminate even rewarded location 5, on the outer array, based on its topological relations to distal environmental cues. The 50% of participants with DS who exhibited above chance performance on AS trials exhibited an intermediate pattern. They showed clear evidence of being able to discriminate the rewarded location 5 on the outer array during both the regular AS trials and the probe trial after a 24-h delay, suggesting the ability to form a long-term topological spatial representation. Their ability to discriminate the other rewarded locations (8 and 10), which require high-resolution spatial coding capacities, was inconsistent during the regular AS trials (**Figure 6**) and was not significant during the probe trial after a 24-h delay (**Figure 8**). However, among those individuals with DS, two participants performed as well as MA-matched TD children, and clearly discriminated the rewarded locations 8 and 10, on the middle and inner arrays, which require the ability to form a high-resolution metric representation of the environment.

Although not entirely conclusive, these preliminary results suggest that important differences exist in the ability of individuals with DS to form different types of AS representations of the environment (topological versus high-resolution metric representations). Moreover, it is also possible that for the participants with DS who failed to show any preference for the rewarded locations on AS trials, the spatial resolution needed to solve the current task was already too high, or that the memory load (three locations) made it too difficult for them to remember even one location using topological information. Systematic investigations of the influence of spatial resolution and memory load are thus required to more firmly establish possible links between dysfunction of specific hippocampal circuits and the AS memory capacities of individuals with DS in large-scale, real world environments.

## CONCLUSION

We have found that individuals with DS could use LCs at a level similar to that of MA-matched TD children in order to identify three rewarded locations among 12 potentially rewarded locations distributed in a 4 m × 4 m square arena. In contrast, in the absence of LCs, when participants must use an allocentric representation of the environment to learn and remember the location of the three rewards, individuals with DS were impaired. This impairment in AS processing stands in contrast to the previously reported preservation of visuospatial capacities demonstrated by individuals with DS on small-scale, egocentrically presented tasks. However, our study also identified important individual variations, with 50% of the participants with DS incapable of any AS learning, 40% capable of better than chance, but less than MA-matched, levels of AS learning, and 10% capable of MA-matched levels of AS learning. These results suggest the existence of identifiable, individual differences in specific hippocampus-dependent memory functions. Future studies should asses the influence of spatial resolution and memory load on the AS memory capacities of individuals with DS in order to investigate potential individual and region-specific hippocampal pathologies.

## AUTHOR CONTRIBUTIONS

Pamela Banta Lavenex and Pierre Lavenex were responsible for the conception and design of the work, acquisition, analysis and interpretation of the data, and drafting of the manuscript. Mathilde Bostelmann, Floriana Costanzo, Emilie Fragnière, Giuliana Klencklen and Deny Menghini were responsible for acquisition and analysis of the data. Catherine Brandner and Stefano Vicari were responsible for conception of the work. All authors have critically revised the mansucript for publication, and agreed to be accountable for all aspects of the work.

## Conflict of Interest Statement

The authors declare that the research was conducted in the absence of any commercial or financial relationships that could be construed as a potential conflict of interest.
